# Effect of amino functional groups on the surface properties and Lewis's acid base parameters of UiO-66(NH_2_) by inverse gas chromatography

**DOI:** 10.1016/j.heliyon.2023.e23839

**Published:** 2023-12-17

**Authors:** Ali Ali-Ahmad, Tayssir Hamieh, Thibault Roques-Carmes, Mohamad Hmadeh, Joumana Toufaily

**Affiliations:** a Laboratory of Materials, Catalysis, Environment and Analytical Methods Laboratory (MCEMA), Faculty of Sciences, Lebanese University, Hadath, Lebanon; b Laboratory of Applied Studies to the Sustainable Development and Renewable Energies (LEADDER), EDST, Faculty of Sciences, Lebanese University, Hadath, Lebanon; c Faculty of Science and Engineering, Maastricht University, P.O. Box 616, 6200 MD Maastricht, the Netherlands; d Université de Lorraine, Laboratoire Réactions et Génie des Procédés, UMR 7274 CNRS, 54000 Nancy, France; e Department of Chemistry, American University of Beirut, P.O. Box 11-0236, Riad El-Solh 1107 2020, Beirut, Lebanon

**Keywords:** MOF, London dispersive component of the surface energy, Specific variables of surface, Lewis acid base parameters

## Abstract

Amino-functionalized metal organic frameworks (MOFs) have attracted much attention for various applications such as carbon dioxide capture, water remediation and catalysis. The focus of this study is to determine the surface and Lewis's acid-base properties of UiO-66(NH_2_) crystals by the inverse gas chromatography (IGC) technique at infinite dilution. The latter was applied to evaluate the dispersive component of the surface energy $\gamma_{s}^{d}\left(T\right)$ by using thermal model and several molecular models. The obtained results proved that $\gamma_{s}^{d}\left(T\right)$ decreases when the temperature increases. The best results were achieved by using the thermal model that takes into account the effect of the temperature on the surface areas of the organic molecules. We also observed a decrease of the Gibbs surface free energy of adsorption by increasing the temperature of the different organic solvents. The polar interactions of UiO-66(NH_2_) were obtained by using the methods of Saint-Flour Papirer, Donnet et al., Brendlé-Papirer and the different molecular models. The Lewis's acid base constants $K_{A}$ and $K_{D}$ were further calculated by determining the different variables of adsorption of the probes on the solid surface and the obtained values were 1.07 and 0.45 for $K_{A}$ and $K_{D}$ respectively, with an acid-base ratio (*K*_*A*_*/K*_*D*_) of 2.38. These values showed the high acidic surface of the solid substrate; whereas, the values of the entropic acid base parameters, $\omega_{A}$, $\omega_{D}$ and $\omega_{A}/\omega_{D}$ respectively equal to $1.0\times 10^{-3}$, $3.8\times 10^{-4}$ and 2.73, also highlighted the important acidity of UiO-66-(NH_2_) surface. These important findings suggest that the surface defects (missing linkers and/or clusters) in UiO-66(NH_2_) are the main determining factor of the acid-base properties of UiO-66 based structures.

## Highlights

• Several IGC methods and models were used to determine $\gamma_{s}^{d}$ of a UiO-66(NH2).
• Hamieh model taking into account the thermal effect gave the more precise results.
• The London dispersive surface energy and specific free variables were determined.
• The UiO-66(NH2) surface exhibited higher acceptor character.
• The addition of amine groups in UiO-66 decreased the surface energy but increased the acid-base behavior.

## Graphical abstract

**Figure:**
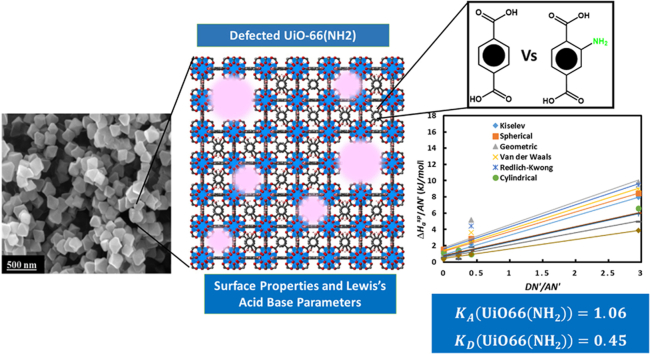

## 1 Introduction

Metal-orgnaic frameworks (MOFs) are constructed by combining metal ions and organic linkers to form porous extended networks of different topologies [1,2]. The wide range of metal nodes and organic linkers, as well as the possibility of post-modifications, have resulted in the development of hundreds of thousands of MOFs structures [3]. Due to their unique characteristics, such as permanent porosity, controllable pore size and functionalization, good chemical and mechanical stability, MOFs have been employed as adsorbents [4], catalysts [[5], [6], [7]], supercapacitors [8] and drug delivery vehicles [9]. As adsorbents, MOFs showed high adsorption capacities for gaseous molecules (e.g. carbon dioxide, methane and hydrogen) [10], heavy metals (e.g. mercury, cadmium and lead) [11] and the separation of gases and other contaminants from diﬀerent environments [12,13].

Among the large number of reported MOF structures, UiO-66 which is based on hexanuclear [Zr_6_O_4_(OH)_4_]^12+^ clusters connected to twelve other clusters via the bridging terephthalate linkers, is one of the most thermally and chemically stable MOFs [14]. UiO-66 and its functionalized derivatives have gained a lot of attention and were used in heterogeneous catalysis, gas separation and storage and water remediation. One of the derivatives, UiO-66(NH_2_), which incorporates amine units in the backbone of the framework showed enhanced adsorption and catalytic properties compared to UiO-66 [[15], [16], [17], [18]]. In addition to the amine functional groups, these UiO-based structures are characterized by the presence of structural defects which originate from the addition of monocarboxylates acids employed as modulators during the crystal synthesis. The modulators which compete with the organic linkers could be removed from the clusters through thermal activation, leading to the formation of structural defects. The latter demonstrated positive impact on the properties of UiO-66 structures, especially in applications such as catalysis and adsorption [19,20].

Therefore, investigating the characteristics of the surfaces of these frameworks such as the nature and distribution of functional groups, defects, surface energy and morphology is necessary to understand their behavior when they are in contact with gases, liquids or other environments. Molecular interactions at the surface of the solid are linked to the surface physicochemical properties which can be analyzed by the determination of the wettability and the calculation of the surface energy via values of contact angles of liquids deposited on the surface [21], calorimetry of adsorption and immersion of solids in a liquid medium [22], adsorption gas and the interpretation of adsorption isotherms whether obtained by static or dynamic methods. Spectroscopic methods, whether infrared, solid-state NMR or electron spectroscopy (ESCA) provide also information about a surface layer of a certain thickness [23,24].

An interesting field of application of MOF materials is their use in different types of chromatography, such as liquid [25] and gas phase chromatography [26,27]. For example, the separation of different mixtures of analytes, such as those of xylene isomers [28], n-alkanes [29], polychlorinated biphenyls [30], polycyclic aromatic hydrocarbons and branched alkanes [31] by MOF-based capillary GC has been demonstrated. Recently, MOFs crystals were employed as the stationary phase in inverse gas chromatography (IGC) at infinite dilution. The aim was not the separation process of the analytes but to study the physico-chemical and surface properties of the MOF through its interaction with probe molecules of different physical and chemical properties [32]. Indeed, IGC at infinite dilution helped in the determination of the surface energy parameters, in addition to the London dispersive surface energy and specific free variables through the injection of probe molecules at infinite dilution of different polarities and topologies [33].

In this study, IGC at infinite dilution was employed to investigate the surface properties of UiO-66(NH_2_) crystals. This involved examining the dispersive surface energy of UiO-66(NH_2_), investigating the specific interactions with polar probes, and estimating the Lewis acid-base parameters of the UiO-66(NH_2_) structure. Furthermore, the obtained data were compared with those previously calculated for UiO-66 to understand the effect of the amine groups as well as the defect number on their surface energy and acid-base behavior.

## 2 Methodology

### 2.1 Materials

Zirconium chloride (ZrCl_4_, 98 %) and acetic acid (C_2_H_4_O_2_, 99 %) were purchased from Acros Organics. 2-Aminoterephthalic acid (C_8_H_7_NO_4_, 99 %) was purchased from Sigma Aldrich. The organic solvents at highly pure grade (99 %) were purchased from Fisher Scientiﬁc.

### 2.2 General synthesis procedure of the UiO-66(NH_2_) based MOFs

The synthesis of UiO-66-(NH_2_) particles was obtained by dissolving 617 mg of 2-Aminoterephthalic acid (3.4 mmol) and 795 mg of Zirconium chloride ZrCl_4_ (3.4 mmol) in 250 mL of dichloromethane (DCM). The mixture was placed in a 500 mL autoclavable reagent bottle before being placed in a sonicator at room temperature. 15 mL of acetic acid was added to the resulting mixture before being placed back into the sonicator. After homogenization of the mixture, the bottle was tightly closed and placed in a preheated oven at 120 °C for 21 h. The obtained solution was then transferred to a falcon tube and the yellow precipitate was collected by centrifugation. The resulting MOF crystals was washed by dimethylformamide (DMF) for five consecutive times over three days then exchanged with DCM for three days. After removing the DCM by centrifugation and washing, the UiO-66-(NH_2_) particles were putted in a vacuum oven at 150 °C overnight for thermal activation.

The obtained crystals were characterized by PXRD, SEM, TGA and BET techniques and compared with previously reported samples of UiO-66-(NH_2_) [34].

### 2.3 Methods of inverse gas chromatography

The methods used in IGC are the same used in another study on UiO-66 [34] such as: the dispersive and non-dispersive parameters of adsorption based on Fowkes relation and developed by Dorris-Gray and Hamieh model, and the methods of Saint-Flour Papirer, Donnet et al. and Brendlé-Papirer. By using the above methods and molecular models, we were able to estimate the dispersive surface energy of UiO-66(NH_2_) surface, the Gibbs free energy of solvents adsorbed on the solid particles, the specific variables of adsorption and the Lewis enthalpic and entropic acid base constants of UiO-66(NH_2_).

## 3 Results and discussion

### 3.1 Structural characterization of UiO-66(NH_2_) catalyst

**Fig. 1:**
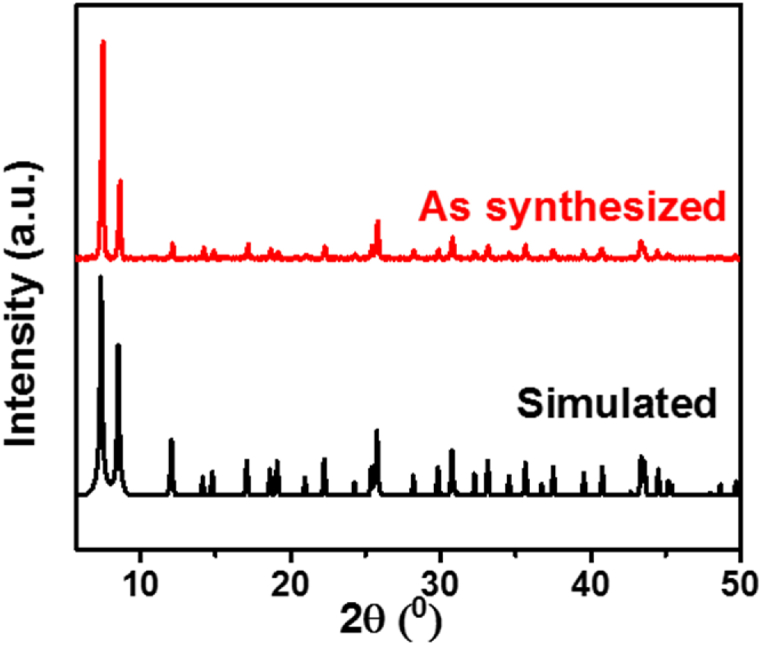
Experimental and simulated PXRD patterns of UiO-66(NH_2_).

The PXRD pattern of the synthesized UiO-66(NH_2_) nanocrystals was recorded and compared to the simulated pattern (Fig. 1). It showed narrow and sharp peaks that are in good agreement with the calculated one, which demonstrates the high crystallinity and the phase purity of the synthesized MOF.

**Fig. 2:**
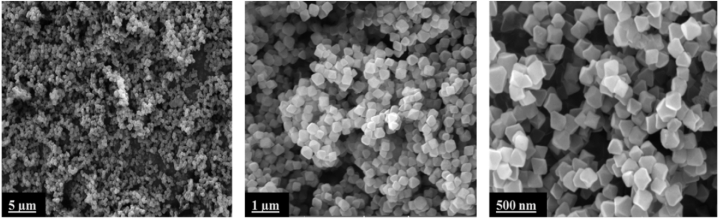
SEM images of UiO-66(NH_2_) at different magnifications.

The SEM images of the synthesized crystals revealed also that UiO-66(NH_2_) sample was pure with homogeneous truncated octahedral shaped crystals of around 100 nm. This crystal shape is typical for UiO-based MOF structures (Fig. 2) [14,34].

**Fig. 3:**
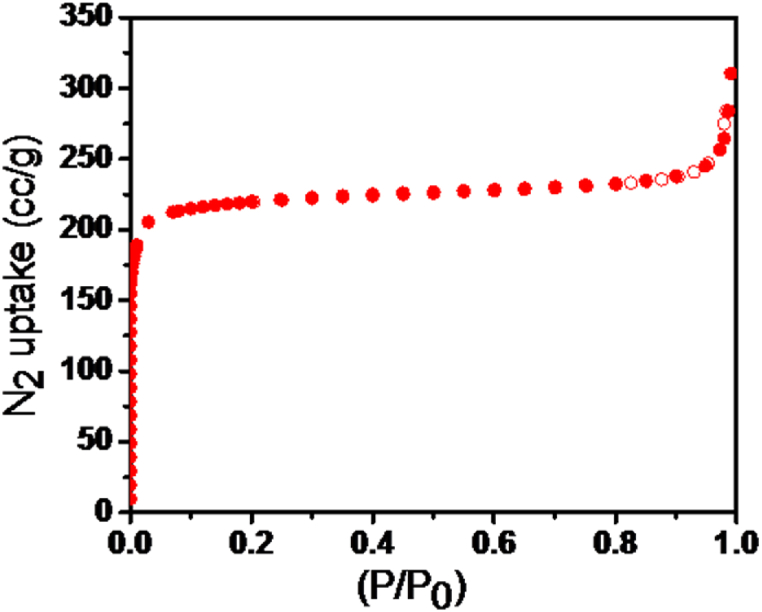
N_2_ adsorption-desorption isotherm of UiO-66(NH_2_) crystals at 77 K.

The nitrogen sorption isotherm of the activated MOF showed an isotherm of type I which is consistent with the microporous nature of MOFs and depicting a monolayer adsorption on their surface (Fig. 3). The calculated Brunauer–Emmett–Teller (BET) surface area was 703 m^2^/g and the pore volume was 0.478 cm^3^/g, which are in agreement with the reported values and lower than those of non-functionalized UiO-66 crystals [35]. This is probably due to the amine groups of the linker that are blocking the pores as it can be seen in the pore size distribution which reveals that the functionalized UiO-66 has smaller pore sizes compared to the non-functionalized (Fig. S1) [36].

**Fig. 4:**
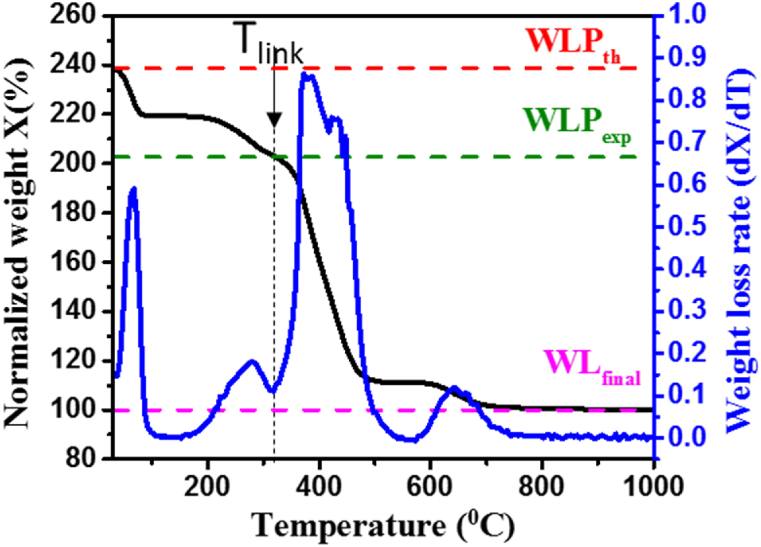
TGA and DTG curves of UiO-66(NH_2_).

The thermogravimetric analysis (TGA) curve of UiO-66(NH_2_) was measured and it showed (Fig. 4). Three phases of weight loss could be distinguished. The first weight loss occurs approximately between 35 °C and 100 °C, where the adsorbed water on the surface of the MOF is volatized. The second weight loss is usually attributed to the removal of the monocarboxylate ligands and to the dehydroxylation of the zirconium clusters, and it extends from 100 °C till T_link_ indicated in Fig. 4. T_link_ is the temperature after which the weight loss is attributed to the combustion of the linker. The third major weight loss in the TGA curve is assigned to the destruction of the framework of the MOF by the combustion of the organic linker. The change in the mass of the sample is attributed to the combustion of the linker which is determined and measured against the theoretical one. The difference between the theoretical and experimentally estimated mass loss is attributed to the presence of defects in the structure.

In this method, it is assumed that 6(ZrO_2_) is the only solid combustion product obtained for UiO-66 and their functionalized version. The combustion of the standard UiO-66 samples is given in the following equation:

$${\text{Zr}}_{6}O_{4}{\left(\text{OH}\right)}_{4}{\left(\text{BDC}\right)}_{6}\left(s\right)+45\;O_{2}\left(g\right)\to 6\;{\text{ZrO}}_{2}\left(s\right)+48\;{\text{CO}}_{2}\left(g\right)+12\;H_{2}O\;\left(g\right)$$

Theoretically, the weight loss plateau is the ratio of the molar mass of the hydroxylated UiO-66 to that of the 6 ZrO_2_. We first start by Normalizing the TGA curve to get a final weight percent at the end equal to 100 %, then the theoretical weight loss could be calculated given the following formula:

$${WLP}_{th}=\frac{{MW}_{MOF}}{{MW}_{6\;{ZrO}_{2}}}*{WL}_{final}$$

where.

${WLP}_{th}$*:* is the theoretical weight loss plateau of the studied hydroxylated MOF structure.

${MW}_{6\;{ZrO}_{2}}$: is the molecular weight of 6 ZrO_2_ (g/mol).

${WL}_{final}$: is the final value of the weight-loss which is set to be 100 % in the normalized curve.

However, the experimental weight loss plateau ${WLP}_{\exp}$ is the horizontal line that passes through the intercept between the TGA curve and vertical line at the temperature indicated *T*_*link*_*. T*_*link*_ is the temperature after which the weight loss is attributed to the combustion of the linker. The value for ${WLP}_{\exp}$ is thus obtained experimentally from the TGA results.

The theoretical weight loss attributed to one linker ${WL}_{link}$ is the difference between the theoretical weight loss plateau and the final weight loss obtained divided by the theoretical number of linkers in the cluster. ${\text{WL}}_{\text{link}}$ is thus calculated given the following formula:

$${WL}_{link}=\frac{{WLP}_{th}-{WL}_{final}}{{NL}_{th}}$$

Where ${NL}_{th}$ is the theoretical number of linkers per hydroxylated Zr_6_ unit.

Since the theoretical weight loss attributed to each linker is known, the actual number of linkers ${NL}_{\exp}$ could be calculated as the ratio of the experimental and theoretical weight losses attributed to the linker, which is expressed as follows:

$${NL}_{\exp}=\frac{{WLP}_{\exp}-{WL}_{final}}{{WL}_{link}}$$

The number of missing linkers, ${NL}_{mis.}$ is then expressed as the difference between the theoretical number of linkers ${NL}_{th}$, and the experimental number of linkers ${NL}_{\exp}$.

$${NL}_{mis.}={NL}_{th}-{NL}_{\exp}$$

The number of missing linkers per cluster was estimated to be 1.56, which is higher than what we obtained for our previously reported UiO-66. All the characteristics extracted from the TGA, BET and SEM analysis were summarized in Table 1 and compared with our previously studied non-functionalized UiO-66 [35].

**Table 1:** Characteristics of the UiO-66 and Ui0-66(NH_2_) crystals.

	Number of missing Linkers	Surface area (m^2^/g)	Particle size (nm)	Pore volume (cc/g)
UiO-66	1.2	988	480	0.512
UiO-66(NH_2_)	1.56	703	230	0.478

### 3.2 Surface properties of UiO-66(NH_2_) catalyst surface by IGC

#### 3.2.1 Gas chromatograph conditions

The experimental conditions of the IGC technique used in this study are similar to that used in our previous study [34]. The column was filled by 170 mg of UiO-66(NH_2_) solid particles. The gas flow rate was optimized at 30 mL/min. The column temperatures were 220–270 °C, varied in 5 °C steps. The net retention volume was calculated by using the classical thermodynamical relations.

#### 3.2.2 The specific free enthalpy of adsorption

Two methods were used in literature to determine the free enthalpy of adsorption $\left(-\Delta G^{0}\right)$ of adsorption of organic solvents on the solid surfaces. They are represented by their reference states: Kemball and Rideal state [36] and De Boer et al. state [37]. In this study, we used the first state of Kemball and Rideal. The specific variables of adsorption such as the specific free enthalpy, enthalpy and entropy of adsorbed molecules on UiO-66(NH_2_) were determined in the temperature interval [493.15K, 543.15K] with the help of molecular models and IGC methods.

#### 3.2.3 The dispersive surface energy of UiO-66(NH_2_)

The methods used to estimate the dispersive surface energy of UiO-66(NH_2_) were based on the Fowlkes's classic relation. Nine methods were used: two based on Dorris-Gray relation, one used our model and six methods used the various molecular models of the surface area of n-alkanes. Hamieh et al. method [38,39] took into account the molecular models of n-alkanes and polar molecules as well as the variations of the surface area as a function of the temperature.

**Fig. 5:**
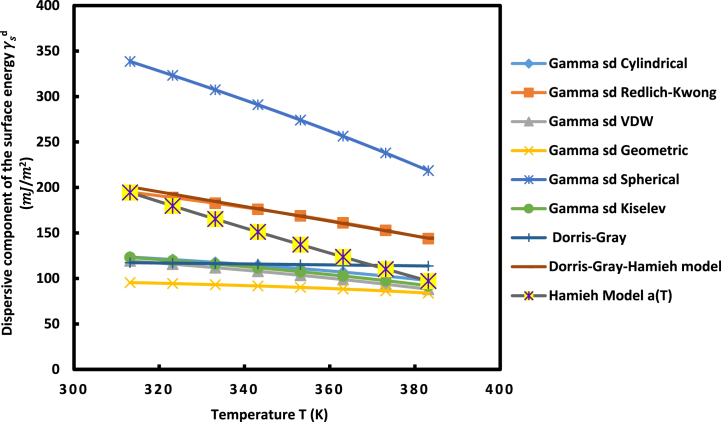
Evolution of $\gamma_{s}^{d}\;\left(mJ/m^{2}\right)$ of UiO-66(NH_2_) versus the temperature for the various IGC methods and models.

The above methods and models were applied to determine the values of $\gamma_{s}^{d}\;\left(T\right)$ of UiO-66(NH_2_) powder at different temperatures (Fig. 5). The curves of Fig. 5 proved a decreasing variation of the dispersive surface energy of UiO-66(NH_2_) solid particles against the temperature.

The more accurate model used for the determination of $\gamma_{s}^{d}\;\left(T\right)$ is that proposed by Hamieh et al. [35]. The results of Fig. 5 showed that the curve representing Hamieh model can be considered as the average mean curve proving the important effect of the temperature on the surface area of organic solvents.

The linear variations of $\gamma_{s}^{d}\left(T\right)$ were given on Table 2, satisfying the following relation:

$$\gamma_{s}^{d}\left(T\right)=a\;T+b$$

Where a and b are respectively given by: $a=\frac{{d\gamma }_{s}^{d}}{dT}$ and ${b=\gamma }_{s}^{d}\left(T=0K\right)$.

**Table 2:** Relations of $\gamma_{s}^{d}\left(T\right)$ of UiO-66(NH_2_) against the temperature with the values of $\varepsilon_{s}^{d}$ and $\gamma_{s}^{d}\left(T=0K\right)$ for the various methods and models.

Molecular model	$\gamma_{s}^{d}\left(T\right)$ (in mJ/m^2^)	$\frac{{d\gamma }_{s}^{d}}{dT}$ (in mJ m^−2^ K^−1^)	$\gamma_{s}^{d}\left(T=0K\right)$ (in mJ/m^2^)
**Cylindrical**	$\gamma_{s}^{d}\left(T\right)$ = −0.3661 T + 239.31	−0.3661	239.31
**Redlich-Kwong**	$\gamma_{s}^{d}\left(T\right)$ = −0.7253 T + 423.68	−0.7253	423.68
**VDW**	$\gamma_{s}^{d}\left(T\right)$ = −0.4434 T + 259.24	−0.4434	259.24
**Geometric**	$\gamma_{s}^{d}\left(T\right)$ = −0.1638 T + 147.47	−0.1638	147.47
**Spherical**	$\gamma_{s}^{d}\left(T\right)$ = −1.7097 T + 876.13	−1.7097	876.13
**Kiselev**	$\gamma_{s}^{d}\left(T\right)$ = −0.4421 T + 262.67	−0.4421	262.67
**Dorris-Gray**	$\gamma_{s}^{d}\left(T\right)$ = −0.0516 T + 133.51	−0.0516	133.51
**Dorris-Gray-Hamieh**	$\gamma_{s}^{d}\left(T\right)$ = −0.807 T + 453.49	−0.807	453.49
**Hamieh Model a(T)**	$\gamma_{s}^{d}\left(T\right)$ = −1.3896 T + 628.7	−1.3896	628.7

The values of the dispersive surface entropy $\frac{{d\gamma }_{s}^{d}}{dT}=\varepsilon_{s}^{d}$ of UiO-66(NH_2_) vary from model to another. The largest value was obtained with the spherical model that also gave the highest value of the extrapolated dispersive surface energy $\gamma_{s}^{d}\left(T=0K\right)$.

**Fig. 6:**
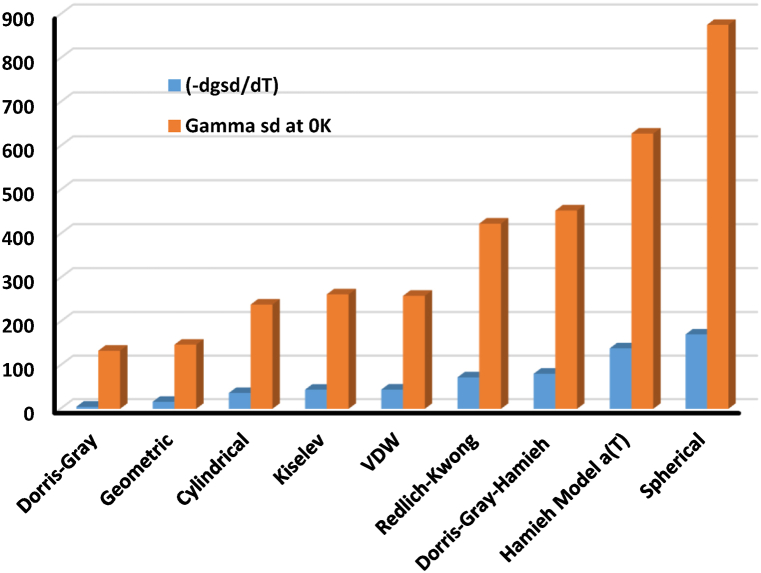
Values of $\gamma_{s}^{d}\left(0K\right)$ and $\frac{{d\gamma }_{s}^{d}}{dT}$ of UiO-66(NH_2_) using the various models.

On Fig. 6, The values of $\frac{{d\gamma }_{s}^{d}}{dT}$ and $\gamma_{s}^{d}\left(0K\right)$ of UiO-66(NH_2_) showed similar increase between their respective representative curves. The lowest values were obtained for Gray method and geometric model; whereas, the highest values were observed with the spherical model that overestimated the surface energy for the different used models. The highest values of $\gamma_{s}^{d}\left(0K\right)$ and $\frac{{d\gamma }_{s}^{d}}{dT}$ (in absolute value) are obtained successively for models taking into account the thermal effect such as Redlich-Kwong model and Hamieh models. The deviation of the spherical model is certainly due to the fact of the overestimation of the surface are of molecules.

Fig. 5, Fig. 6 and Table 2 showed closer similarity between Redlich-Kwong, Hamieh and Dorris-Gray-Hamieh models. In fact, these three models used the thermal effect on the surface areas of n-alkanes with more accurate estimation when using Hamieh model which determined more accurately the surface areas of molecules. By applying Hamieh model, we obtained the variations of $\gamma_{s}^{d}\left(T\right)$ of UiO-66(NH_2_) particles:

$$\gamma_{s}^{d}\left(T\right)=-1.390\;T+628.7$$

#### 3.2.4 Determination of the specific free energy and acid-base properties of UiO-66(NH_2_) particles

On Tables S1–S10, we gave the obtained variations of the specific free energy ($\Delta G_{a}^{sp}\left(T\right)$) of adsorption of the polar molecules on UiO-66(NH_2_) surface by using the three methods of Brendlé-Papirer [40], Donnet et al. [41] and Saint-Flour-Papirer [42] and the other models [39].

**Fig. 7:**
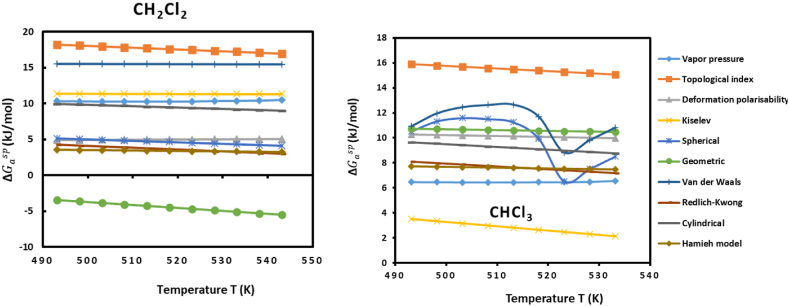
Variations of $\Delta G_{a}^{sp}$ of dichloromethane adsorbed on UiO-66 (NH_2_) particles against the temperature for the various models or methods (left). Variations of $\Delta G_{a}^{sp}$ of chloroform adsorbed on UiO-66 (NH_2_) particles against the temperature for the various models or methods (right).

Tables S1–S10 allowed to obtain the linear relations of the specific free enthalpy ($\Delta G_{a}^{sp}\left(T\right)$) as a function of the temperature relative to the various polar molecules by using the different IGC models and methods. The values of ($\Delta G_{a}^{sp}\left(T\right)$) presented on these Tables, at a fixed temperature, vary from one model to another. These variations can be in certain models three times higher than the other methods or models. The curves plotted on Fig. 7 for dichloromethane and chloroform showed the large difference between the values of the specific free energy of an organic probe when the applied model changed.

IGC is considered to be an excellent technique to characterize the solid substrates, however, large differences between the obtained values of the specific parameters of adsorption of polar solvents on a solid surface at fixed temperature were observed. In fact, only Hamieh model proved its validity in the determination of both the dispersive surface energy and specific variables of adsorption. This is due to the fact that the thermal effect on the surface area was taken into account by this model. In literature, many scientists neglected the effect of the temperature on the value of the surface area of organic molecules and consequently, the determination of the specific free enthalpy and the dispersive surface energy of materials were not accurate. In our study, Hamieh model was demonstrated to be the most accurate method followed successively by the methods of Saint-Flour Papirer, Donnet et al. and Brendlé-Papirer.

#### 3.2.5 Enthalpic acid base constants

In order to compare between the various IGC methods used in this study, the values of the specific enthalpy ($-\Delta H_{a}^{sp})$ and specific entropy of adsorption ($-\Delta S_{a}^{sp})$ of organic molecules on UiO-66(NH_2_) solid particles were given in Table 3, Table 4.

**Table 3:** Values of ($-\Delta H_{a}^{sp}\;in\;kJ\;{mol}^{-1}$) of adsorption of polar molecules on UiO-66 (NH_2_) surface material by comparing between the various methods and the global average.

Model or method	CH2Cl2	Chloroform	Toluene	Benzene
**Kiselev**	20.45	11.50	1.31	26.30
**Spherical**	29.14	15.00	1.70	27.91
**Geometric**	14.20	17.30	3.10	32.43
**Van der Waals**	19.71	16.84	2.17	29.60
**Redlich-Kwong**	19.50	16.57	2.65	31.22
**Cylindrical**	20.50	19.69	0.56	21.77
**Hamieh model**	10.78	6.50	1.45	19.50
**Topological index**	15.38	17.61	0.84	19.88
**Deformation polarizability**	10.10	5.01	1.10	16.50
**Vapor pressure**	6.49	10.32	0.55	12.78
**Global average**	16.63	13.63	1.54	23.79

**Table 4:** Values of ($-\Delta S_{a}^{sp}in\;J\;K^{-1}{mol}^{-1}$) of adsorption of polar molecules on UiO-66 (NH_2_) surface material by comparing between the various methods and the global average.

Model or method	CH2Cl2	Chloroform	Toluene	Benzene
**Kiselev**	34	23	3	50
**Spherical**	52	20	3	66
**Geometric**	7	42	5	78
**Van der Waals**	24	3	1	27
**Redlich-Kwong**	23	25	1	38
**Cylindrical**	22	20	1	32
**Hamieh model**	6	6	1	19
**Topological index**	0	0	0	0
**Deformation polarizability**	7	3	1	19
**Vapor pressure**	0	0	0	0
**Average value**	17	14	2	33

These results gave different values of the specific enthalpy and entropy according to the used IGC method. The same difficulties previously encountered with the values of the specific free enthalpy were also observed here with the values of ($-\Delta H_{a}^{sp})$ and ($-\Delta S_{a}^{sp})$. The previous conclusion about the accurate results of Hamieh model is also valid in this case.

**Fig. 8:**
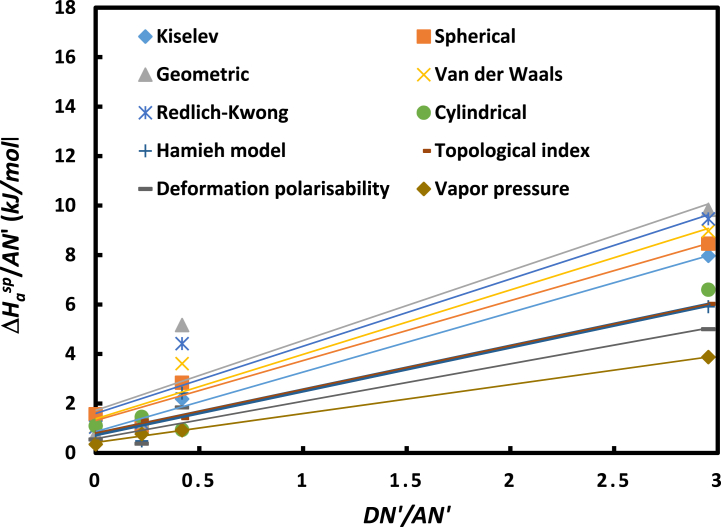
Variations of $\left(\frac{-\Delta H_{a}^{sp}}{{AN}^{\prime }}\right)$ as a function of $\left(\frac{{DN}^{\prime }}{{AN}^{\prime }}\right)$ for the methods.

**Fig. 9:**
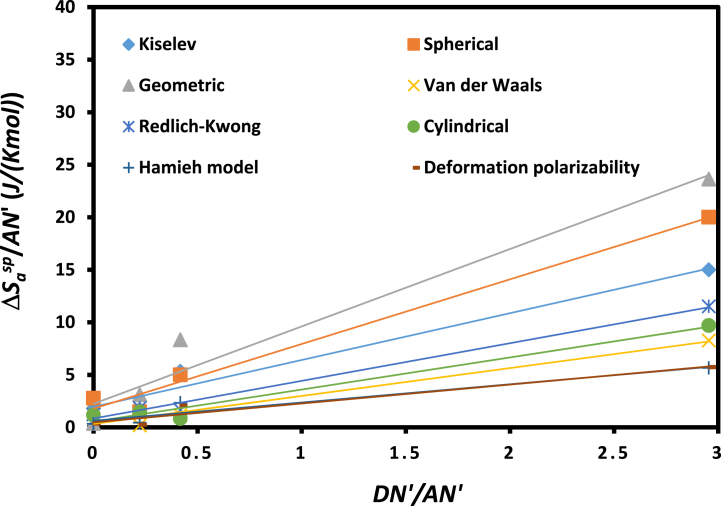
Variations of $\left(\frac{-\Delta S_{a}^{sp}}{{AN}^{\prime }}\right)$ as a function of $\left(\frac{{DN}^{\prime }}{{AN}^{\prime }}\right)$ for the various methods.

The large differences between the obtained values of ($-\Delta H_{a}^{sp})$ and ($-\Delta S_{a}^{sp})$ depending on the chosen method or model led to different values of the Lewis acid-base constants and then different characteristics for the same solid substrates. To clarify this, the evolution of $\left(\frac{-\Delta H_{a}^{sp}}{{AN}^{\prime }}\right)$ and $\left(\frac{-\Delta S_{a}^{sp}}{{AN}^{\prime }}\right)$ as a function of $\left(\frac{{DN}^{\prime }}{{AN}^{\prime }}\right)$ were plotted in Fig. 8, Fig. 9 for the various methods or models.

The obtained results were summarized in Table 5 and showing the different values of the enthalpic acid base constants $K_{A}$ and $K_{D}$ and entropic acid base parameters $\omega_{A}$ and $\omega_{D}$ according to the used IGC method.

**Table 5:** Values of Lewis's acid base parameters $K_{A}$ and $K_{D}$ (enthalpic) and the entropic acid base constants $\omega_{A}$ and $\omega_{D}$ (entropic) of UIO-66 (NH_2_) solid particles and their ratios depending to the chosen method.

Models and IGC methods	*K*_*A*_	*K*_*D*_	*K*_*A*_*/K*_*D*_	*ω*_*A*_	*ω*_*D*_	*ω*_*A*_*/ω*_*D*_
Kiselev	1.44	0.52	2.79	2.7 × 10^−3^	1.2 × 10^−3^	2.27
Spherical	1.45	0.78	1.85	3.7 × 10^−3^	1.1 × 10^−3^	3.44
Geometric	1.69	1.03	1.64	4.3 × 10^−3^	1.7 × 10^−3^	2.59
Van der Waals	1.56	0.82	1.89	1.6 × 10^−3^	2.0 × 10^−4^	7.79
Redlich-Kwong	1.62	0.95	1.70	2.1 × 10^−3^	5.1 × 10^−4^	4.23
Cylindrical	1.16	0.46	2.52	1.8 × 10^−3^	3.3 × 10^−4^	5.60
Hamieh model	1.06	0.45	2.35	1.0 × 10^−3^	3.8 × 10^−4^	2.73
Topological index	1.05	0.48	2.20	1.1 × 10^−3^	2.9 × 10^−4^	3.76
Deformation polarizability	0.90	0.34	2.62	2.3 × 10^−3^	7.0 × 10^−4^	3.26
Vapor pressure	0.70	0.25	2.75	2.7 × 10^−3^	1.2 × 10^−3^	2.27
Global average	1.26	0.61	2.07	3.7 × 10^−3^	1.1 × 10^−3^	3.44

Table 5 proved the higher acidic character of UiO-66(NH_2_) crystals in Lewis terms according to Hamieh model. These values are in agreement with the previous work on this defected MOF as catalyst for esterification reactions which require an acidic behavior of the MOF surface [[35], [36], [37],^43^]. The defected nature of this MOF was also highlighted by the TGA analysis showing 1.56 missing linkers per cluster resulted in the creation of Lewis acid sites on the Zr-clusters. The acidic character of UiO-66(NH_2_) was also proved by all IGC used methods or models with an advantage to Hamieh model taking into account the thermal effect.

#### 3.2.6 Comparison with UiO-66

In a previous study [35], Hamieh model was employed to estimate the specific parameters, Lewis Acid-Base constants and $\gamma_{s}^{d}\left(T\right)$ of UiO-66. The obtained equation $\gamma_{s}^{d}\left(T\right)$ of UiO-66 solid surface is given below against the temperature:

$$\gamma_{s}^{d}\left(T\right)\left(\text{UiO}-66\right)=-0.444\;T+190.86$$

Hamieh et al. [35] also showed the acidic character of UiO-66 surface and determined its acid and base constants:

$$K_{A}\left(\text{UiO}-66\right)=0.57\;\text{and}\;K_{D}\left(\text{UiO}-66\right)=0.18$$

In this study, we proved that the presence of amine groups in UiO-66 decreased the specific surface area, pore volume and particle size, but also increased the number of defects in cluster. The determination of $\gamma_{s}^{d}\left(T\right)$ of UiO-66(NH_2_) by using Hamieh model gave the following relation:

$$\gamma_{s}^{d}\left(T\right)\;\left(\text{UiO}-66\left({\text{NH}}_{2}\right)\right)=-1.390\;T+628.7$$

By comparing the two MOFs UiO-66 and UiO-66(NH_2_), It can be clearly noticed that the surface energy of UiO-66(NH_2_) is greater than that for UiO-66 for all molecular models used. The introduction of NH_2_ groups in the backbone of the UiO-66 structure resulted in an increase in the London dispersive surface energy.

The constants $K_{A}$ and $K_{D}$ of UiO-66(NH_2_) were determined:

$$K_{A}\left({\text{UiO}-66(\text{NH}}_{2})\right)=1.06\;\text{and}\;K_{D}\left({\text{UiO}-66(\text{NH}}_{2})\right)=0.45$$

In terms of acidity, we observed that the acid character is greater than the basic character for both MOFs. By comparing the acid-base constants of these two MOFs, we notice that the acid constant for UiO-66(NH_2_) is clearly greater than that of UiO-66. The ratio between the two acid constants is given by:

$$\frac{K_{A}\left(\text{UiO}-66\left({\text{NH}}_{2}\right)\right)}{K_{A}\left(\text{UiO}-66\right)}=1.86$$

The above ratio greater than 1 is certainly due to the fact that the number of defects in the structure of UiO-66(NH_2_) is greater than that in UiO-66, therefore, the number of acid sites in UiO-66(NH_2_) is greater than that in UiO-66, for this we notice that the acidity of UiO-66(NH_2_) is greater than that of UiO-66.

At the level of basicity, we notice that the basic character at the level of UiO-66(NH_2_) is even greater than in UiO-66. The ratio between the two basic constants is given by:

$$\frac{K_{D}\left({\text{UiO}-66(\text{NH}}_{2})\right)}{K_{D}\left(\text{UiO}-66\right)}=2.5$$

This is due to the basic functional group NH_2_ which is present in the structure of UiO-66(NH_2_). The NH_2_ groups of UiO-66(NH_2_) have increased the basicity constant. The Lewis acid base sites of UiO-66(NH_2_) have both increased with respect to UiO-66.

## 4 Conclusions

The specific free energy, enthalpy and entropy of adsorption of polar organic solvents adsorbed on UiO-66(NH_2_) surface were evaluated by using ten different IGC methods and models included Hamieh model that took into account the thermal effect. The seven molecular models were used to determine the dispersive component of the surface energy of UiO-66(NH_2_) solid particles. The results obtained by applying Hamieh model showed a strong acid character of the used MOF with an acid base ratio greater than 2. The same result was observed with the entropic acid base constant. One obtained the equation of the dispersive surface energy against the temperature:

$$\gamma_{s}^{d}\left(T\right)\;\left(\text{UiO}-66\left({\text{NH}}_{2}\right)\right)=-1.390\;T+628.7$$

The comparison between the obtained results with UiO-66(NH_2_) and that of UiO-66 surface led to conclude that the presence of amine groups in the backbone of the framework decreased the specific surface area, the pore volume and the particle size, but increased the dispersive surface energy and the acid base character of the MOF structure.

## Ethical approval

This article does not contain any studies with human participants performed by any of the authors.

## Data availability statement

Data are included in the article and in supplementary material.

## CRediT authorship contribution statement

**Ali Ali-Ahmad:** Writing – review & editing, Writing – original draft, Validation, Methodology, Investigation, Formal analysis. **Tayssir Hamieh:** Writing – review & editing, Writing – original draft, Validation, Supervision, Resources, Project administration, Methodology, Investigation, Funding acquisition, Formal analysis, Conceptualization. **Thibault Roques-Carmes:** Writing – original draft, Validation, Supervision, Methodology, Investigation, Formal analysis, Conceptualization. **Mohamad Hmadeh:** Writing – review & editing, Writing – original draft, Validation, Supervision, Methodology, Investigation, Funding acquisition, Formal analysis, Conceptualization. **Joumana Toufaily:** Writing – original draft, Validation, Supervision, Resources, Project administration, Investigation, Funding acquisition, Formal analysis, Conceptualization.

## Declaration of competing interest

The authors declare that they have no known competing financial interests or personal relationships that could have appeared to influence the work reported in this paper.
