# Supplementary material for: Effect of amino functional groups on the surface properties and Lewis's acid base parameters of UiO-66(NH2) by inverse gas chromatography
Source: Heliyon. 2023 Dec 17;10(1):e23839. doi: 10.1016/j.heliyon.2023.e23839 (PMC10788446; doi:10.1016/j.heliyon.2023.e23839)
Supplement: Multimedia component 1 [file mmc1.docx]

**Effect of Amino Functional Groups on the Surface Properties and Lewis’s Acid Base Parameters of UiO-66(NH_2_) by Inverse Gas Chromatography**

**Ali Ali-Ahmad^1,2,5^, Tayssir Hamieh^3^**^^[[1]](#footnote-1)^*^, **Thibault Roques-Carmes^4^, Mohamad Hmadeh^5*^, Joumana Toufaily^1,2^**

^1^Laboratory of Materials, Catalysis, Environment and Analytical Methods Laboratory (MCEMA), Faculty of Sciences, Lebanese University, Hadath, Lebanon.

^2^Laboratory of Applied Studies to the Sustainable Development and Renewable Energies (LEADDER), EDST, Faculty of Sciences, Lebanese University, Hadath, Lebanon.

^3^Faculty of Science and Engineering, Maastricht University, P.O. Box 616, 6200 MD Maastricht, The Netherlands.

^4^Université de Lorraine, Laboratoire Réactions et Génie des Procédés, UMR 7274 CNRS, 54000 Nancy, France.

^5^Department of Chemistry, American University of Beirut, P.O. Box 11-0236, Riad El-Solh 1107 2020, Beirut, Lebanon.


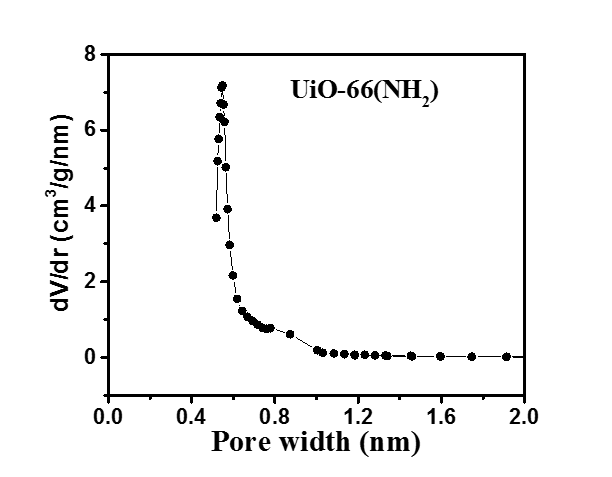


Figure S1: Pore size distribution of UiO-66(NH_2_).

Table S1: Variations of ($\Delta G_{a}^{sp}\left( T \right)$) (in kJ/mol) versus the temperature of the adsorption of polar molecules on UiO-66 (NH_2_) surface material when using Saint-Flour Papirer method.

| T (K) | CH_2_Cl_2_ | Chloroform | Toluene | Benzene |
| --- | --- | --- | --- | --- |
| 493.15 | 10.312 | 6.465 | 5.629 | 0.057 |
| 498.15 | 10.287 | 6.456 | 5.557 | 0.052 |
| 503.15 | 10.264 | 6.447 | 5.484 | 0.047 |
| 508.15 | 10.255 | 6.442 | 5.412 | 0.042 |
| 513.15 | 10.254 | 6.439 | 5.339 | 0.037 |
| 518.15 | 10.270 | 6.455 | 5.267 | 0.032 |
| 523.15 | 10.271 | 6.462 | 5.194 | 0.027 |
| 528.15 | 10.325 | 6.490 | 5.122 | 0.022 |
| 533.15 | 10.351 | 6.577 | 5.049 | 0.017 |
| 538.15 | 10.389 | 6.537 | 4.977 | 0.012 |
| 543.15 | 10.484 | 6.607 | 4.904 | 0.007 |

Table S2: Variations of ($\Delta G_{a}^{sp}\left( T \right)$) (in kJ/mol) versus the temperature of the adsorption of polar molecules on UiO-66 (NH_2_) surface material when using Donnet et al. method.

| T (K) | CH_2_Cl_2_ | Chloroform | Toluene | Benzene |
| --- | --- | --- | --- | --- |
| 493.15 | 18.234 | 15.884 | 3.606 | 3.751 |
| 498.15 | 18.110 | 15.783 | 3.441 | 3.780 |
| 503.15 | 17.986 | 15.683 | 3.276 | 3.810 |
| 508.15 | 17.862 | 15.582 | 3.111 | 3.839 |
| 513.15 | 17.738 | 15.482 | 2.946 | 3.869 |
| 518.15 | 17.614 | 15.381 | 2.781 | 3.898 |
| 523.15 | 17.490 | 15.281 | 2.616 | 3.928 |
| 528.15 | 17.366 | 15.180 | 2.451 | 3.957 |
| 533.15 | 17.242 | 15.080 | 2.286 | 3.987 |
| 538.15 | 17.118 | 14.979 | 2.121 | 4.016 |
| 543.15 | 16.994 | 14.879 | 1.956 | 4.046 |

Table S3: Variations of ($\Delta G_{a}^{sp}\left( T \right)$) (in kJ/mol) versus the temperature of the adsorption of polar molecules on UiO-66 (NH_2_) surface material when using Brendlé and Papirer method.

| T (K) | CH_2_Cl_2_ | Chloroform | Toluene | Benzene |
| --- | --- | --- | --- | --- |
| 493.15 | 4.923 | 10.264 | 7.179 | 0.508 |
| 498.15 | 4.940 | 10.230 | 7.085 | 0.502 |
| 503.15 | 4.956 | 10.197 | 6.990 | 0.496 |
| 508.15 | 4.973 | 10.163 | 6.896 | 0.490 |
| 513.15 | 4.989 | 10.130 | 6.801 | 0.484 |
| 518.15 | 5.006 | 10.096 | 6.707 | 0.478 |
| 523.15 | 5.022 | 10.063 | 6.612 | 0.472 |
| 528.15 | 5.039 | 10.029 | 6.518 | 0.466 |
| 533.15 | 5.055 | 9.996 | 6.423 | 0.460 |
| 538.15 | 5.072 | 9.962 | 6.329 | 0.454 |
| 543.15 | 5.088 | 9.929 | 6.234 | 0.448 |

Table S4: Variations of ($\Delta G_{a}^{sp}\left( T \right)$) (in kJ/mol) versus the temperature of the adsorption of polar molecules on UiO-66 (NH_2_) surface material by applying Kiselev method.

| T (K) | CH_2_Cl_2_ | Chloroform | Toluene | Benzene |
| --- | --- | --- | --- | --- |
| 493.15 | 0.010 | 3.831 | 1.889 | -0.268 |
| 498.15 | -0.107 | 3.662 | 1.642 | -0.284 |
| 503.15 | -0.223 | 3.494 | 1.394 | -0.300 |
| 508.15 | -0.340 | 3.325 | 1.147 | -0.316 |
| 513.15 | -0.456 | 3.157 | 0.899 | -0.332 |
| 518.15 | -0.573 | 2.988 | 0.652 | -0.348 |
| 523.15 | -0.689 | 2.820 | 0.404 | -0.364 |
| 528.15 | -0.806 | 2.651 | 0.157 | -0.380 |
| 533.15 | -0.922 | 2.483 | -0.091 | -0.396 |
| 538.15 | -1.039 | 2.314 | -0.338 | -0.412 |
| 543.15 | -1.155 | 2.146 | -0.586 | -0.428 |

Table S5: Variations of ($\Delta G_{a}^{sp}\left( T \right)$) (in kJ/mol) versus the temperature of the adsorption of polar molecules on UiO-66 (NH_2_) surface material by applying spherical model.

| T (K) | CH_2_Cl_2_ | Chloroform | Toluene | Benzene |
| --- | --- | --- | --- | --- |
| 493.15 | 5.137 | 10.533 | -4.630 | 0.221 |
| 498.15 | 5.037 | 11.329 | -4.960 | 0.206 |
| 503.15 | 4.937 | 11.593 | -5.290 | 0.191 |
| 508.15 | 4.837 | 11.513 | -5.620 | 0.176 |
| 513.15 | 4.737 | 11.262 | -5.950 | 0.161 |
| 518.15 | 4.637 | 9.947 | -6.280 | 0.146 |
| 523.15 | 4.537 | 6.512 | -6.610 | 0.131 |
| 528.15 | 4.437 | 7.512 | -6.940 | 0.116 |
| 533.15 | 4.337 | 8.512 | -7.270 | 0.101 |
| 538.15 | 4.237 | 9.512 | -7.600 | 0.086 |
| 543.15 | 4.137 | 10.512 | -7.930 | 0.071 |

Table S6: Variations of ($\Delta G_{a}^{sp}\left( T \right)$) (in kJ/mol) versus the temperature of the adsorption of polar molecules on UiO-66 (NH_2_) surface material by applying geometric model.

| T (K) | CH_2_Cl_2_ | Chloroform | Toluene | Benzene |
| --- | --- | --- | --- | --- |
| 493.15 | -3.412 | 10.750 | 22.663 | 0.634 |
| 498.15 | -3.622 | 10.715 | 25.070 | 0.609 |
| 503.15 | -3.832 | 10.680 | 27.045 | 0.584 |
| 508.15 | -4.042 | 10.645 | 28.848 | 0.559 |
| 513.15 | -4.252 | 10.610 | 30.583 | 0.534 |
| 518.15 | -4.462 | 10.575 | 31.660 | 0.509 |
| 523.15 | -4.672 | 10.540 | 32.282 | 0.484 |
| 528.15 | -4.882 | 10.505 | 33.282 | 0.459 |
| 533.15 | -5.092 | 10.470 | 34.282 | 0.434 |
| 538.15 | -5.302 | 10.435 | 35.282 | 0.409 |
| 543.15 | -5.512 | 10.400 | 36.282 | 0.384 |

Table S7: Variations of ($\Delta G_{a}^{sp}\left( T \right)$) (in kJ/mol) versus the temperature of the adsorption of polar molecules on UiO-66 (NH_2_) surface material by applying van der Waals model.

| T (K) | CH_2_Cl_2_ | Chloroform | Toluene | Benzene |
| --- | --- | --- | --- | --- |
| 493.15 | 15.554 | 10.937 | 16.137 | 1.821 |
| 498.15 | 15.541 | 11.957 | 16.001 | 1.818 |
| 503.15 | 15.528 | 12.457 | 15.864 | 1.814 |
| 508.15 | 15.515 | 12.632 | 15.728 | 1.811 |
| 513.15 | 15.502 | 12.668 | 15.591 | 1.807 |
| 518.15 | 15.489 | 11.704 | 15.455 | 1.804 |
| 523.15 | 15.476 | 8.828 | 15.318 | 1.800 |
| 528.15 | 15.463 | 9.828 | 15.182 | 1.797 |
| 533.15 | 15.450 | 10.828 | 15.045 | 1.793 |
| 538.15 | 15.437 | 11.828 | 14.909 | 1.790 |
| 543.15 | 15.424 | 12.828 | 14.772 | 1.786 |

Table S8: Variations of ($\Delta G_{a}^{sp}\left( T \right)$) (in kJ/mol) versus the temperature of the adsorption of polar molecules on UiO-66 (NH_2_) surface material by applying Redlich-Kwong method.

| T (K) | CH_2_Cl_2_ | Chloroform | Toluene | Benzene |
| --- | --- | --- | --- | --- |
| 493.15 | 4.245 | 8.109 | 12.480 | 2.157 |
| 498.15 | 4.120 | 7.994 | 12.290 | 2.152 |
| 503.15 | 3.995 | 7.878 | 12.100 | 2.147 |
| 508.15 | 3.870 | 7.763 | 11.910 | 2.142 |
| 513.15 | 3.745 | 7.647 | 11.720 | 2.137 |
| 518.15 | 3.620 | 7.532 | 11.530 | 2.132 |
| 523.15 | 3.495 | 7.416 | 11.340 | 2.127 |
| 528.15 | 3.370 | 7.301 | 11.150 | 2.122 |
| 533.15 | 3.245 | 7.185 | 10.960 | 2.117 |
| 538.15 | 3.120 | 7.070 | 10.770 | 2.112 |
| 543.15 | 2.995 | 6.954 | 10.580 | 2.107 |

Table S9: Variations of ($\Delta G_{a}^{sp}\left( T \right)$) (in kJ/mol) versus the temperature of the adsorption of polar molecules on UiO-66 (NH_2_) surface material by applying cylindrical method.

| T (K) | CH_2_Cl_2_ | Chloroform | Toluene | Benzene |
| --- | --- | --- | --- | --- |
| 493.15 | 9.975 | 9.654 | 5.986 | 0.314 |
| 498.15 | 9.876 | 9.544 | 5.826 | 0.312 |
| 503.15 | 9.778 | 9.434 | 5.666 | 0.309 |
| 508.15 | 9.679 | 9.324 | 5.506 | 0.307 |
| 513.15 | 9.581 | 9.214 | 5.346 | 0.304 |
| 518.15 | 9.482 | 9.104 | 5.186 | 0.302 |
| 523.15 | 9.384 | 8.994 | 5.026 | 0.299 |
| 528.15 | 9.285 | 8.884 | 4.866 | 0.297 |
| 533.15 | 9.187 | 8.774 | 4.706 | 0.294 |
| 538.15 | 9.088 | 8.664 | 4.546 | 0.292 |
| 543.15 | 8.990 | 8.554 | 4.386 | 0.289 |

Table S10: Variations of ($\Delta G_{a}^{sp}\left( T \right)$) (in kJ/mol) versus the temperature of the adsorption of polar molecules on UiO-66 (NH_2_) surface material when using by applying by using Hamieh model.

| T (K) | CH_2_Cl_2_ | Chloroform | Toluene | Benzene |
| --- | --- | --- | --- | --- |
| 493.15 | 3.541 | 7.722 | 10.278 | 0.760 |
| 498.15 | 3.511 | 7.691 | 10.185 | 0.753 |
| 503.15 | 3.481 | 7.660 | 10.091 | 0.746 |
| 508.15 | 3.451 | 7.629 | 9.998 | 0.739 |
| 513.15 | 3.421 | 7.598 | 9.904 | 0.732 |
| 518.15 | 3.391 | 7.567 | 9.811 | 0.725 |
| 523.15 | 3.361 | 7.536 | 9.717 | 0.718 |
| 528.15 | 3.331 | 7.505 | 9.624 | 0.711 |
| 533.15 | 3.301 | 7.474 | 9.530 | 0.704 |
| 538.15 | 3.271 | 7.443 | 9.437 | 0.697 |
| 543.15 | 3.241 | 7.412 | 9.343 | 0.690 |

1. ***Corresponding authors:***

   Tayssir Hamieh, Email: [t.hamieh@maastrichtuniversity.nl](mailto:t.hamieh@maastrichtuniversity.nl), <https://orcid.org/0000-0003-2153-7408>), Faculty of Science and Engineering, Maastricht University, P.O. Box 616, 6200 MD Maastricht, The Netherlands.

   Mohamad Hmadeh, Email: [mohamad.hmadeh@aub.edu.lb](mailto:mohamad.hmadeh@aub.edu.lb), American University of Beirut, P.O. Box 11-0236, Riad El-Solh 1107 2020, Beirut, Lebanon.  [↑](#footnote-ref-1)
